# Functional limitations and firearm purchases among low-income U.S. military veterans

**DOI:** 10.1186/s40621-024-00553-x

**Published:** 2024-12-18

**Authors:** Alexander Testa, Jack Tsai

**Affiliations:** 1 Department of Management, Policy and Community Health, University of Texas Health Science Center at Houston, 1200 Pressler Street, Houston, TX 77030 USA; 2 Department of Veterans Affairs, National Center on Homelessness Among Veterans, Washington, DC USA

**Keywords:** Functional Limitations, Firearms, Veterans, Veteran Affairs

## Abstract

**Objectives:**

Functional limitations may be associated with firearm ownership among veterans by amplifying perceptions of vulnerability and the need for security, yet their role remains underexplored despite emerging research on health-related factors influencing firearm acquisition in this group. This study examines the relationship between the number of functional limitations and recent firearm purchasing among a sample of low-income US military veterans.

**Methods:**

Data are from the National Veteran Homeless and Other Poverty Experiences (NV-HOPE) study, which surveyed US veterans in households under 300% of the US federal poverty level. The survey was conducted in December 2022 and January 2023 (*n* = 1,008). Functional limitations were assessed based on self-reported assistance needed in daily tasks. Multiple logistic regression was used to analyze the association between functional limitations and firearm purchases in the past year, adjusting for demographic and socioeconomic factors. Analyses were conducted in Stata v. 18, and statistical significance was determined at the *p* < 0.05 threshold.

**Results:**

5.5% of respondents reported purchasing a firearm in the past year. Increased functional limitations were positively associated with recent firearm purchases (Adjusted Odds Ratio [aOR] = 1.14, 95% Confidence Interval [CI] = 1.03, 1.26). Sensitivity using Firth Logit for rare events confirmed the robustness of this finding.

**Discussion:**

Veterans experiencing a greater number of functional limitations are more likely to report recently purchasing a firearm. These findings underscore the importance of addressing health needs among firearm-owning veterans through VA programs that support disabled veterans and their caregivers.

## Introduction

The United States (US) has an estimated 400 million firearms in circulation [11], with levels of firearm purchasing surging considerably in recent years, particularly in the aftermath of the COVID-19 pandemic [18] and purchasing remaining at record highs in 2023–2024 [8]. Given this considerable increase in firearm ownership, understanding the motivations behind firearm acquisition has become critically important. This is particularly the case among certain segments of the population, such as US military veterans, who represent a unique group of firearm owners both due to their formal training in and the use of firearms [12, 24], but also their elevated rates of suicide—the majority of which are firearm related [22, 29].

While individuals purchase firearms for various reasons, including personal protection and recreational purposes [3, 14], any relationship between firearm purchases and one’s health has been understudied. Indeed, many veterans experience functional limitations—i.e., restrictions that prevent a person from performing tasks or activities [16, 17]. Functional limitations may be associated with perceived vulnerability and reduced physical autonomy, heightening the desire for security and control, which firearm ownership may seem to offer veterans [5, 19]. As such, functional limitations could serve as a strong motivator for firearm acquisition in this group, reflecting both a psychological need for protection and a coping mechanism amid physical decline. While emerging research has begun to explore how health-related factors are associated with patterns of firearm ownership and firearm acquisition among veterans [5, 21, 23], the role of functional limitations has gone overlooked.

In this study, we analyze national data from low-income US military veterans collected from December 2022 and January 2023 to examine the relationship between functional limitations and recent firearm purchases. This study offers an important contribution considering the elevated prevalence of functional limitations among veteran populations [16], the focus of the US Department of Veterans (VA) healthcare system on reducing morbidity and promoting functional independence [17], and the risk for firearm-related suicides in the veteran population [22, 29], as well as functional limitations as a suicide risk-factor [7, 10]. Using observational cross-sectional data from a survey of US veterans, we hypothesize that veterans who self-report a greater number of functional limitations will be more likely to report having purchased a firearm in the past year.

## Methods

### Data

The data used in the current study are from the National Veteran Homeless and Other Poverty Experiences (NV-HOPE) study, an series of nationally representative surveys led by the VA National Center on Homelessness among Veterans for the purposes of studying the health and social determinants of health among low-income US veterans [23]. NV-HOPE utilized a large, probability-based representative online panel of the US population from the Ipsos KnowledgePanel^®^ that includes over 70,000 households. The sampling approach included address-based sampling using the Ipsos KnowledgePanel^®^, where active panel members were weighted according to demographic distributions from the latest US Census Bureau’s Current Population Survey. Ipsos employed a probability-proportional-to-size method to select participants. All data collection adhered to Ipsos’s institutional security and human subjects protocols, with informed consent obtained from all respondents and a de-identified dataset provided to the research team.

Data collection took place between December 13, 2022, and January 6, 2023. Selected panel members received an email invitation to complete the survey and respondents completed s creening survey to confirm their eligibility criteria. The 1,028 veterans who met these criteria completed the survey, which took approximately 45 min. Eligibilty for the NV-HOPE study were veterans aged 18 and older, who had served on active duty in the military, and were living in households with incomes under 300% of the federal poverty level as of 2022. In 2022, the federal poverty level threshold was $14,880 for one person, $18,900 for two people, and $23,280 for three people, and the national poverty rate was 11.5% [28]. In the NV-HOPE study, data on household income were provided to the research team through a self-reported survey question inquiring about pre-tax earnings. Respondents selected income levels from a range of intervals, starting at less than $5,000 and extending to $250,000 or higher. To create a continuous income variable, the midpoint of each income interval was used. Subsequently, the reported household income was adjusted for household size using the equivalence scale method [27], calculated as: adjusted income = household income / (household size^0.5). The sample had an average adjusted household income of $32,788 and an adjusted median household income of $26,515.

### Dependent variable

*Purchased firearm in the past year* is a variable based on a question asking, “In the past year, have you acquired any new guns or revolvers?” (yes or no) [21].

### Independent variable

*Functional Limitations* is measured using a scale created from 11 items that respondents answered either “yes” or “no”. Specifically, respondents are asked, “At the present time, do you need help from another person to do any of the following? (1) to bathe (wash or dry your whole body), (2) to walk around your home or apartment, (3) to dress (like putting on a shirt or shoes, buttoning, or zipping), (4) to get in and out of a chair, (5) shop for groceries, clothing, or other items, (6) go to the doctor, (7) travel to visit friends, go to church or temple, etc., (8) pay bills or manage money, (9) prepare meals, (10) do household chores (e.g., laundry, cleaning), (11) take medication properly. Items were summed into a scale ranging from 0–11 (Kuder-Richardson Coefficient = .904) [9].

### Control variables

Control variables are included measuring respondents’ demographic, socioeconomic status, and household-related characteristics, which prior research suggests may be associated with veteran’s health status, as well as patterns of firearm ownership and acquisition [5, 21]. Control variables include respondent’s *age* in years, *biological sex* (female or male), *race/ethnicity* (White, Black, Other Race, or Hispanic), *marital status* (not married or married), household *income* (<$24,999, $25,000 to $49,999, $50,000 to $74,999, or ≥$75,000), *educational attainment* (less than college, or college graduate), *number of persons in the household* (1, 2, 3, or 4+), *children in the household* (no or yes), *housing type* (single-family detached, single family condo/townhouse, apartment, or other), and *military branch* (Army, Air Force, Marine Corps, Navy, Coast Guard, or Reserves).

### Statistical analysis

First, we display the summary statistics of the analytic sample. Next, we assess the bivariate relationship between functional limitations and a firearm purchase in the past year with a two-tailed t-test. Then, we assessed the covariate-adjusted relationship using multiple logistic regression, with coefficients converted to odds ratios. We then follow up on those analyses with a supplementary set of analyses using the Firth method of the rare event logistic regression model to address small-sample bias stemming from rare outcomes through a penalized likelihood approach [4, 6]. Standard errors are clustered on state of residence. Analyses were conducted in Stata v.18, and statistical significance is determined at *p* < 0.05 threshold. Due to small cell sizes because of the frequency of the outcome variable, subgroup or interaction analyses were not conducted.

## Results

**Fig. 1:**
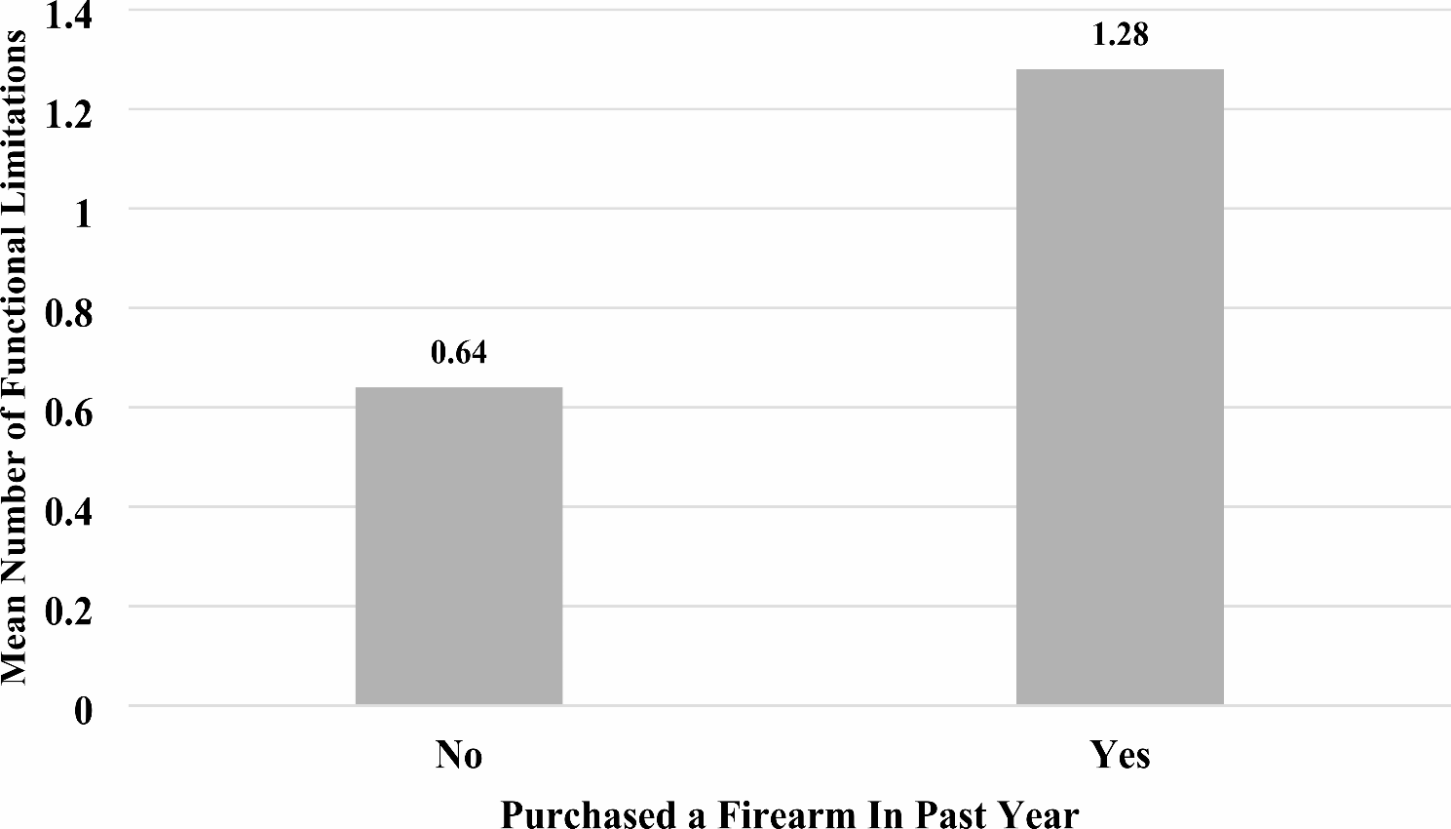
Mean Number of Functional Limitations by Past Year Firearm Purchasing (*N =* 1,008). *Note: *Results of a two-tailed *t-*test reveal that the difference in the mean number of functional limitations by past year firearm purchasing is statistically significant (t-statistic= 2.252, *p *= .025)

Of the 1,028 original survey respondents, 2% of respondents were missing data on the variable measuring firearm purchase in the past year, resulting in a final analytic sample including 1,008 respondents. Table 1 presents the summary statistics of the analytic sample, showing that 5.5% of respondents reported purchasing a firearm in the past year. The functional limitations variable ranged from 0 to 11 with a mean score of 0.67 (standard deviation = 1.94). The sample had a mean age of approximately 65 years, is 89.7% male, and is mostly non-Hispanic White (72.8%), with fewer respondents identifying as non-Hispanic Black (13.8%), Hispanic (8.1%) or other race/ethnicity (5.3%). Figure 1 shows that the average number of functional limitations among veterans was significantly higher among those who purchased a firearm in the past year (1.28) compared to 0.64 among those who did not purchase a firearm in the past year (*t*-statistic = 2.252, *p* = 0.025).

**Table 1:** Summary Statistics of Analytic Sample (*N* = 1,008)

Variable	Unweighted Frequency	Weighted %/ Mean (SD)
*Purchased Firearm in Past Year*		
No	953	94.5
Yes	55	5.5
Functional Limitations	1,008	0.67 (1.94)
Age	1,008	65.17 (15.65)
*Biological Sex*		
Female	123	10.3
Male	885	89.7
*Race/Ethnicity*		
White	777	72.8
Black	102	13.8
Other Race	48	5.3
Hispanic	81	8.1
*Marital Status*		
Not Married	461	46.8
Married	547	53.2
*Income*		
<$24,999	288	30.3
$25,000 to $49,999	511	48.2
$50,000 to $74,999	158	16.2
≥$75,000	51	5.4
*Educational Attainment*		
Less than College	753	82.5
College Graduate	255	17.5
*Number of Persons in Household*		
1	320	30.2
2	462	43.6
3	113	10.9
4+	113	15.2
*Children in Household*		
No	884	82.8
Yes	124	17.2
*Housing Type*		
Single Family Detached	690	67.9
Single Family Condo or Townhouse	64	6.0
Apartment Building	172	17.6
Other	82	8.5
*Military Branch*		
Army	369	38.3
Air Force	250	23.5
Marine Corps	63	7.2
Navy	195	18.3
Coast Guard	61	6.1
Reserves	70	6.6

Table 2 presents the results of the multiple logistic regression model, showing that there is a positive association between the number of functional limitations and having purchased a firearm in the past year (Adjusted Odds Ratio [aOR] = 1.14, 95% Confidence Interval [CI] = 1.03, 1.26). Findings from the Firth logit model in Appendix A provide consistent results, with the number of functional limitations retaining a positive and statistically significant association with having purchased a firearm in the past year (aOR = 1.13, 95% CI = 1.01, 1.26).

**Table 2:** Multiple Logistic Regression of Purchased Firearm in Past Year on Functional Limitations and Other Covariates (*N* = 1,008)

Variables	OR	95% CI
Functional Limitations	1.14*	(1.03–1.26)
Age	0.99	(0.97–1.01)
Male	1.88	(0.53–6.63)
White (Reference)	—	—
Black	2.02	(0.97–4.23)
Other Race	0.40	(0.09–1.83)
Hispanic	0.07*	(0.01–0.86)
Married	1.37	(0.56–3.33)
<$24,999 (Reference)	—	—
$25,000 to $49,999	0.89	(0.32–2.43)
$50,000 to $74,999	1.34	(0.58–3.10)
≥$75,000	1.70	(0.40–7.23)
College Graduate	1.77	(0.90–3.47)
Household Size − 1 (Reference)	—	—
Household Size − 2	0.53	(0.20–1.39)
Household Size − 3	0.21*	(0.05–0.82)
Household Size − 4+	1.32	(0.50–3.47)
Children in Household	1.85	(0.76–4.50)
Single Family Detached (Reference)	—	—
Single Family Condo or Townhouse	0.04**	(0.00–0.36)
Apartment Building	0.17*	(0.04–0.69)
Other	1.71	(0.59–4.93)
Army (Reference)	—	—
Air Force	1.66	(0.81–3.40)
Marine Corps	1.07	(0.35–3.28)
Navy	0.40	(0.16–1.06)
Coast Guard	0.91	(0.21–3.89)
Reserves	0.94	(0.24–3.66)

***p < 0.001, **p < 0.01, *p < 0.05

Because the measure of functional limitations includes measures that can be considered parts of separate constructs, including activities of daily living and instrumental activities, of daily living, additional analyses were conducted that separate the functional limitations variable into two separate variables composed of items that are *activities of daily living*: needing help to (a) bathe, (b) walk around home or apartment, (c) dress, and (d) get in and out of a chair, and items that are *instrumental activities of daily living*: needing help to (a) shop for groceries, (b) go to the doctor, (c) travel to visit friends, go to church or temple, etc., (d) pay bills or manage money, (e) prepare meals, (f) do household chores or (g) take medication properly. The results from separate multiple logistic regressions in Appendix B show that both activities of daily living (aOR = 1.46, 1.06, 2.01) and instrumental activities of daily living (aOR = 1.18, 95% CI = 1.02, 1.35) are positively and significantly associated with a recent firearm purchase.

## Discussion

The findings from the current study demonstrate an association between a greater number of functional limitations and recent firearm acquisition among veterans. This relationship aligns with prior research indicating that functional impairments can amplify feelings of vulnerability and a need for self-protection [1]. In the context of veterans, who often have higher rates of functional limitations [16, 17], the need for personal safety may be heightened, which could be associated with firearm purchases as a means of coping with functional limitations. This is consistent with research on firearms and threat sensitivity, which suggests that individuals may turn to firearms to feel secure in the face of perceived risks, even if this perception is not directly related to actual safety threats [19].

These findings also raise important considerations for public health interventions targeting veterans with functional limitations. Given that firearm access is associated with an increased risk of suicide, especially among veterans [22, 29], and that functional limitations also increase suicide risk [7, 10], programs that support veterans in managing functional limitations through VA healthcare system initiatives could benefit from incorporating risk-reduction strategies for firearm ownership, including counseling on secure storage and alternative ways to bolster personal security and autonomy [2, 13].

There may also be opportunities for VA and community partners to develop further programming to help veterans with functional limitations achieve feelings of safety and security. For example, the Veteran Directed Care program offers veterans a budget to hire caregivers, including family members, to assist them with daily living activities [25]. The VA Caregiver Support Program [26] also exists to provide resources for caregivers of eligible veterans, and caregivers may be important stakeholders in assisting veterans with their physical limitations as well as safe storage of firearms and monitoring suicide risk [15]. By enabling veterans to hire caregivers and receive personalized assistance with daily living, these programs can mitigate feelings of vulnerability and reduce the perceived need for firearms as a source of security. Additionally, caregivers involved in these programs can play a critical role in promoting safe firearm storage practices and monitoring mental health risks, including suicide prevention. Integrating firearm safety counseling and secure storage education into these initiatives could enhance their impact.

### Limitations and directions for future research

This study has limitations that should be considered when interpreting the results. First, the focus on low-income military veterans who are, on average, age 65 potentially limits the generalizability of the study findings. Future work that examines the relationship between functional limitations and firearm purchasing among broader samples of the veteran population, as well as among the general population, would be valuable. Second, the firearm purchasing variable is limited to recent purchases within the past year. Future research should incorporate more comprehensive measures of firearm acquisition, including detailed life history data on the timing of firearm purchases and their relationship to significant life events, such as the onset of functional limitations or changes in health status. Additionally, capturing information on the types and quantities of firearms purchased would provide valuable insights into patterns of firearm ownership and their potential links to health-related factors.

Third, because of the sample size and the total number of respondents reporting firearm purchases within the past year limit, this study could not analyze differences in the observed associations by age, race, gender, or socioeconomic status. Fourth, the measure of functional limitations did not include data on the timing of when the limitation began or the severity of the limitation, which may impact firearm acquisition behavior. Additionally, the current study lacks data on the reasons or motivations underpinning firearm purchasing behavior. A valuable direction for future research would be to collect more detailed data about the timing, severity, and subjective perceptions of functional limitations, as well as reasons for why a firearm was purchased to understand how and why functional limitations are associated with firearm acquisition behavior through longitudinal data that can better track changes in health and firearm purchasing over time. Finally, it is possible that information on firearm purchasing may be under-reported due to hesitancy to disclose firearm ownership or that individuals may underreport their functional limitation status due to social desirability bias in favor of better health. Thus, it may be valuable for future research to compare findings from self-reported data with available administrative data, such as Veteran Health Administration records, to determine how changes in health status correspond with firearm purchasing behavior.

## Conclusions

The results of this national study indicate that veterans with a greater number of functional limitations are more likely to report purchasing a firearm in the past year. Given the elevated rates of suicide within the US veteran population [22, 29] and the increased risk associated with both functional limitations [16, 17] and firearm ownership [20] concerning suicidal behaviors, these findings underscore the importance of further exploring the factors driving firearm purchasing among veterans.

## Appendix A: Firth Multiple Logistic Regression of Purchased Firearm in Past Year on Functional Limitations and Other Covariates (*N* = 1,008)

**Table:** 

Variables	OR	95% CI
Functional Limitations	1.13*	(1.01–1.26)
Age	0.98*	(0.95–1.00)
Male	2.46	(0.87–6.95)
White (Reference)	—	—
Black	1.60	(0.69–3.70)
Other Race	0.69	(0.20–2.39)
Hispanic	0.23	(0.04–1.25)
Married	1.29	(0.57–2.92)
—	—	
$25,000 to $49,999	1.15	(0.56–2.37)
$50,000 to $74,999	1.55	(0.61–3.92)
≥$75,000	1.28	(0.35–4.68)
College Graduate	1.02	(0.53–1.94)
Household Size − 1 (Reference)	—	—
Household Size − 2	0.48	(0.19–1.23)
Household Size − 3	0.37	(0.10–1.36)
Household Size − 4+	1.29	(0.39–4.35)
Children in Household	1.11	(0.42–2.90)
Single Family Detached (Reference)	—	—
Single Family Condo or Townhouse	0.28	(0.05–1.60)
Apartment Building	0.34	(0.11–1.00)
Other	2.04	(0.90–4.58)
Army (Reference)	—	—
Air Force	1.31	(0.67–2.57)
Marine Corps	0.74	(0.22–2.47)
Navy	0.73	(0.31–1.74)
Coast Guard	0.99	(0.30–3.34)
Reserves	1.10	(0.37–3.23)

***p < 0.001, **p < 0.01, *p < 0.05

## Appendix B: Multiple Logistic Regression of Purchased Firearm in Past Year on Activities of Daily Living and Instrumental Activities of Daily Living (*N* = 1,008)

**Table:** 

Variables	OR	95% CI
Activities of Daily Living	1.46*	(1.06–2.01)
Instrumental Activities of Daily Living	1.18*	(1.02–1.35)

*p < 0.05

Control variables include: age, biological sex, race/ethnicity, marital status, income, educational attainment, number of persons in the household, children in the household, housing type, and military branch

*Note*: each row represents separate multiple logistic regression models

## Data Availability

Data are available upon reasonable request from the authors.
